# Retraction: Comparing the controlled attenuation parameter using FibroScan and attenuation imaging with ultrasound as a novel measurement for liver steatosis

**DOI:** 10.1371/journal.pone.0273606

**Published:** 2022-08-22

**Authors:** The PLOS ONE Editors

After this article [1] was published, it came to light that the study design, patient information, and attenuation imaging (ATI) results reported in [1] were published previously in [2]. There is also substantial text overlap between the two articles. The earlier publication [2] evaluated the accuracy of ATI in evaluating liver steatosis, whereas the *PLOS ONE* article [1] compared the accuracy of ATI and controlled attenuation parameter in evaluating liver steatosis.

This issue was discussed with the authors. The first author acknowledged that the same cohort of patients and ATI data is reported in both articles [1,2]. While the articles address different research questions, the reuse of content was not acknowledged in [1]. Hence, the article does not adhere to the journal’s second publication criterion or PLOS’ Editorial policies pertaining to text reuse and publication of related studies, and concerns remain regarding partial redundancy of the two articles [1,2].

The ethics approval number (210202) in [1] is also reported in several articles by some of the same authors, including [3–7], which describe studies and patient cohorts different to those reported in [1]. Additionally, discrepancies were identified between the description of the study design in [1] and the ethics approval documentation provided during discussions.

The first author stated that the same approval number (210202) was obtained for multiple different studies [1,3–7] as the applications for approval for all the studies were made to the IRB together. They also acknowledged that the study described in [1] was retrospective, which conflicts with the description of the study design in [1]. The editors remain concerned regarding the approval of multiple different studies together and that the study design is not accurately reported in [1].

Due to the concerns regarding the reuse of text and data, the *PLOS ONE* Editors retract this article.

WWS, PYS, and CLW agreed with the retraction. WWS stands by the article’s findings. CLW apologizes for the issues with the published article. PKH, LSW, YYC, YCH, and HHY either did not respond directly or could not be reached.
